# Tetralone derivatives are MIF tautomerase inhibitors and attenuate macrophage activation and amplify the hypothermic response in endotoxemic mice

**DOI:** 10.1080/14756366.2021.1916010

**Published:** 2021-07-06

**Authors:** János Garai, Marcell Krekó, László Őrfi, Péter Balázs Jakus, Zoltán Rumbus, Patrik Kéringer, András Garami, Eszter Vámos, Dominika Kovács, Viola Bagóné Vántus, Balázs Radnai, Tamás Lóránd

**Affiliations:** a Department of Pathophysiology, Institute for Translational Medicine, University of Pécs, Medical School, Pécs, Hungary; b Department of Pharmaceutical Chemistry, Semmelweis University, Budapest, Hungary; c Department of Biochemistry and Medical Chemistry, University of Pécs, Medical School, Pécs, Hungary; d Department of Thermophysiology, Institute for Translational Medicine, University of Pécs, Medical School, Pécs, Hungary

**Keywords:** MIF, MIF inhibitors, 2-arylmethylene-1-tetralones, tautomerase, inflammation

## Abstract

Macrophage migration inhibitory factor (MIF) is a pro-inflammatory cytokine playing crucial role in immunity. MIF exerts a unique tautomerase enzymatic activity that has relevance concerning its multiple functions and its small molecule inhibitors have been proven to block its pro-inflammatory effects. Here we demonstrate that some of the *E*-2-arylmethylene-1-tetralones and their heteroanalogues efficiently bind to MIF’s active site and inhibit MIF tautomeric (enolase, ketolase activity) functions. A small set of the synthesised derivatives, namely compounds (**4**), (**23**), (**24**), (**26**) and (**32**), reduced inflammatory macrophage activation. Two of the selected compounds (**24**) and (**26**), however, markedly inhibited ROS and nitrite production, NF-κB activation, TNF-α, IL-6 and CCL-2 cytokine expression. Pre-treatment of mice with compound (**24**) exaggerated the hypothermic response to high dose of bacterial endotoxin. Our experiments suggest that tetralones and their derivatives inhibit MIF’s tautomeric functions and regulate macrophage activation and thermal changes in severe forms of systemic inflammation.

## Introduction

Macrophage migration inhibitory factor (MIF) was the first representative of those polypeptide immune mediators that have been grouped later as “cytokines”^1^^,^^2^. Since its description 50 years ago MIF has accumulated a bewildering variety of immune and non-immune functions^3^. MIF has been also considered to be a missing link between inflammation and tumorigenesis^4^.

MIF structure shares no homology with other known cytokines. Its structural relatives are the mammalian enzyme, d-dopachrome-tautomerase (DDT)^5^, and the prokaryotic enzymes: chorismate mutase, 5-carboxymethyl-2-hydroxymuconate-isomerase (CHMI), *trans*- and *cis*-3-chloroacrylic acid dehalogenase (CaaD and *cis*-CaaD, respectively), and 4-oxalocrotonate-tautomerase (4-OT)^6^^,^^7^. MIF exists in a homotrimeric form of 12.4 kD monomers. In each monomer, two α-helices are packed against a four-stranded β-sheet^8^. There are reports, however, of the occurrence of a dimeric form of MIF^9^ as well as of other oligomerisation states^10^ and also of heteromers^11^.

More than twenty years ago Rorsman et al. reported that recombinant MIF catalyses the tautomerisation of the non-naturally occurring d-dopachrome, transforming the coloured compound to the colourless dihydroindole carboxylic acid (DHICA)^12^, however, l-dopachrome methyl ester (a melanin precursor) have also been found a suitable substrate. This was soon followed by the identification of phenylpyruvate and *p*-hydroxyphenylpyruvate as alternate substrates for the tautomeric activity of MIF^13^. In the homotrimeric MIF, the catalytic site is located between each of two adjacent monomers. Acidic p*K*a of the *N*-terminal proline of each MIF monomer is thought to play a crucial role in the keto-enol tautomerisation reaction^7^. A direct link between cytokine activity and tautomerase catalytic site of MIF has been reported^14^, although a “true” endogenous small molecule ligand has yet to be found. Blocking of endogenous MIF by a small molecule such as “ISO-1” and neutralisation of MIF by anti-MIF antibodies or by plant-derived MIF inhibitors reduces the manifestations of inflammatory conditions such as type II collagen-induced arthritis, immunologically induced kidney disease, experimental autoimmune encephalomyelitis, experimental allergic neuritis, immunoinflammatory diabetes, experimental autoimmune myocarditis, irradiation-induced acute pneumonitis, sepsis, and ischemia–reperfusion injury^15–18^. Therefore, inhibitors of MIF tautomerase hold promise for prospective clinical use in many pathologic conditions^19^. Development of individualised therapy targeting MIF in these conditions is expected based on the human genetic data supporting the role of high-expression MIF alleles in the clinical severity and end-organ complications of a number of inflammatory disorders^20^.

Molecular modelling techniques have been used lately to find “designer” inhibitors of promising potential^21–23^. The reported MIF enzyme inhibitors include long-chain fatty acids, *E-*2-fluoro-*p*-hydroxycinnamate, dopachrome analogues, tryptophan and tyrosine derivatives^24^, or coumarin- and chromenone derivatives^19^^,^^25^.

A large variety of natural compounds show inhibitory effect of the MIF enzyme including e.g. ellagic acid^26^. According to previous results, caffeic acid proved to be an efficient inhibitor of MIF tautomerase when using either dopachrome^27^ or phenylpyruvate^28^ as the substrate. Phenylpyruvate tautomerase was also inhibited by curcumin^28^, a substance known long for its anti-inflammatory and cancer chemopreventive properties – a constituent of the spice turmeric^29^. Therefore, the selection of our potential MIF inhibitors was based on the unsaturated ketone – structural motif – found in several natural or synthetic MIF inhibitors such as coumarines and chromenones and cinnamates such as caffeic acid, curcumin, and *N*-acetyl-*p*-benzoquinone^19^. In addition, according to our previous studies, some tetralone derivatives proved to be efficient MIF inhibitors^30^.

Based on these findings, we have focused on the group of cyclic α,β-unsaturated ketones to study their inhibitory effects towards the MIF tautomerase activity. To find compounds with good inhibitory effect, we have previously selected some different model compounds as 2-arylidenecycloalkanones, 2,5-diarylidene-cyclopentanone and few 2-arylidene-1-tetralones^30^. The enol-keto tautomeric conversion of phenylpyruvate (ketonase reaction) was investigated by spectrophotometric method. From the substances investigated, several proved to be good inhibitors of the tautomerase activity of the MIF enzyme.

This paper deals with a large molecular library of *E*-2-arylmethylene-1-tetralones and their hetero-analogues related to naturally occurring flavonoids. Some representatives of this latter group have previously been proven as potent MIFtautomerase inhibitors^28^^,^^31^. At this end, potential analogies between the biological roles of flavonoids and melanins (or precursors thereof) deserve particular attention^32^ concerning possible implication of MIF in (neuro-) melanogenesis^33^.

**Figure 1.**
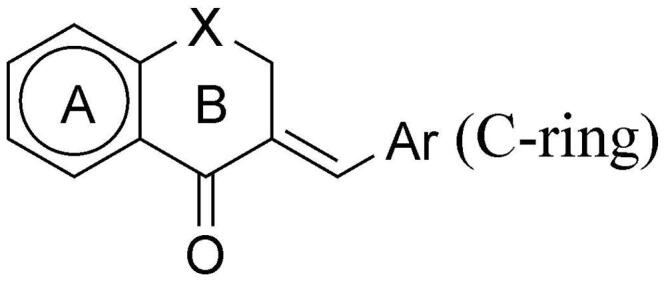
General formula of the test compounds **(1–38)**.

The molecule library we have used here shows large structural diversity. We have chosen cyclic ketones of different size and with different heteroatoms as skeletal compounds, and applied various aryl side chains to find an optimal lead compound. We have varied the size of the ketone ring, the aromatic substituent C-ring (with electron withdrawing and electron donating groups), and also changed the type of the B-ring (see Figure 1 and Table 1). Through *in silico* methods, we aimed to identify the key interactions required for MIF inhibitory activity. In addition, we tested the biological activity of the best tautomerase inhibitors from the molecular library in lipopolysaccharide-(LPS)-induced macrophages and also in LPS-treated mice.

**Table 1.** Inhibitor activity of the test compounds (1–38) in the enol-keto and keto-enol tautomeric conversion of phenylpyruvate.

Compounds	X	Ar	Inhibition of ketonase (IC_50_, μM)	Inhibition of enolase (IC_50_, μM)
**1**	–	Phenyl	82.8 ± 16.5	20.2 ± 3.9
**2**	CH_2_	Phenyl	127 ± 20	113 ± 16
**3**	(CH_2_)_2_	Phenyl	84.0 ± 15.3	129 ± 19
**4**	CH_2_	4′- CH_3_-phenyl	16.5 ± 3.4	2290 ± 343
**5**	CH_2_	4′-OCH_3_-phenyl	57.0 ± 14.1	33.4 ± 8.8
**6**	CH_2_	4′-Cl-phenyl	59.1 ± 9.7	42.8 ± 3.1
**7**	CH_2_	2′-Cl-phenyl	2640 ± 406	458 ± 73
**8**	CH_2_	4′-Br-phenyl	52.2 ± 11.5	232 ± 54
**9**	CH_2_	3′-Br-phenyl	7340 ± 1024	3350 ± 424
**10**	CH_2_	4′-F-phenyl	72.4 ± 3.4	42.1 ± 4.3
**11**	CH_2_	2′,6′-(Cl)_2_-phenyl	58.9 ± 10.3	35.5 ± 5.9
**12**	CH_2_	3′,4′-(Cl)_2_-phenyl	2620 ± 696	91.5 ± 9.0
**13**	CH_2_	2′,4′-(Cl)_2_-phenyl	16.9 ± 4.2	301 ± 42
**14**	CH_2_	4′-COOH-phenyl	187 ± 32	37.7 ± 16.3
**15**	CH_2_	4′-(NCH_3_)_2_-_-_phenyl	90.2 ± 15.1	495 ± 101
**16**	CH_2_	4′-CN-phenyl	146 ± 17	43.2 ± 12.4
**17**	CH_2_	3′,4′-(OCH_3_)_2_ -phenyl	585 ± 94	302 ± 63
**18**	CH_2_	3′,4′-(OCH_2_O)-phenyl	50.7 ± 6.0	213 ± 31
**19**	CH_2_	2′-Furyl	57.7 ± 12.6	25.4 ± 4.7
**20**	CH_2_	2′-Thienyl	54.0 ± 5.5	52.3 ± 7.6
**21**	CH_2_	2′-Pyrrolyl	142 ± 31	125 ± 39
**22**	CH_2_	*N*-Methyl-2′-pyrrolyl	23.8 ± 3.8	27.8 ± 5.6
**23**	CH_2_	3′-Indolyl	58.8 ± 10.6	2.89 ± 0.75
**24**	CH_2_	2′-Pyridyl	5.63 ± 0.94	28.6 ± 7.4
**25**	CH_2_	3′-Pyridyl	20.3 ± 5.5	28.6 ± 6.0
**26**	CH_2_	4′-Pyridyl	21.0 ± 6.9	209 ± 77
**27**	O	Phenyl	267 ± 59	1740 ± 483
**28**	O	4′- CH_3_-phenyl	102 ± 19	3620 ± 829
**29**	O	3′- CH_3_-phenyl	56.1 ± 8.8	443 ± 112
**30**	O	4′-OCH_3_-phenyl	96.0 ± 29.9	425 ± 70
**31**	O	3′-OCH_3_-phenyl	97.0 ± 20.7	205 ± 32
**32**	O	2′-OCH_3_-phenyl	4.31 ± 1.34	1260 ± 159
**33**	O	4′-Br-phenyl	311 ± 77	1890 ± 438
**34**	O	2′-Br-phenyl	98.3 ± 16.0	3200 ± 887
**35**	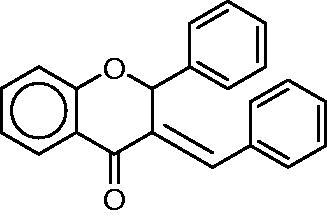		419 ± 113	– (Because of low solubility data are not available)
**36**	S	Phenyl	208 ± 42	633 ± 108
**37**	SO	Phenyl	94.6 ± 25.4	193 ± 59
**38**	SO_2_	Phenyl	160 ± 38	151 ± 27

## Material and methods

### Reagents

**Scheme 1.**
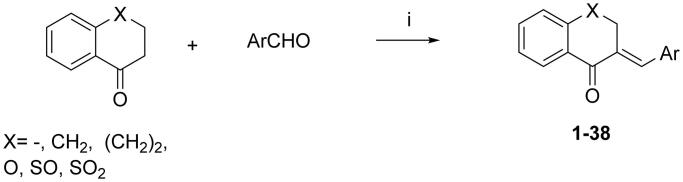
General synthetic method for the preparation of the title compounds **1–38.** Reagents and conditions (i) base as NaOH or piperidine, temperature: ambient temperature or 140 °C.

All reagents were purchased from Sigma-Aldrich (St. Louis, MO) unless otherwise indicated. A molecular library of 38 structurally related known compounds has been studied. Based on their structural features, this library could be further divided into two subgroups (Table 1). The group I consists of a 1-indanone derivative (**1**), substituted arylmethylene–tetralones (**2**, **4–18**), heteroaryl–methylene–tetralones (**19–26**) and a benzosuberone derivative (**3**). Group II contains chromanones and their thioanalogues (**27–38**). All of the compounds investigated here have been prepared by base catalysed aldol condensation, the conditions (temperature, solvent and catalyst) of the synthetic method were a little bit different. See the detailed methods below. (Their structure is depicted in Supplementary material's Figure S1 and in Table 1; the synthesis method is in Ref. [34].) Scheme 1 describes the general synthetic method. The preparation of the *E*-2-phenylmethylene-1-indanone (**1**), *E*-2-phenylmethylene-1-benzosuberone (**3**) and E-2-arylmethylene-1-tetralones (**2, 4–26**) was carried out at room temperature in ethanol. The synthesis of the *E*-3-arylmethylenechroman-4-ones and *E*-3-arylmethylene-1-thiochroman-4-ones (**27–36**) was performed at 140 °C with piperidine as a catalyst without solvent. The 4-thiochromanone-1-oxide (**36**) and −1,1-dioxide (**37**) was obtained according to literature methods^35^. All of the compounds were purified by recrystallisation from methanol and with column chromatography. Their structural characterisation is based on FT-IR methods and previously published NMR data^36–53^. Thus, all of the test compounds studied here have been published elsewhere and characterised, see the references above. All of the compounds were proved as *E*-isomer based on the NMR measurements^54^.

### Tautomerase assay

The enol-keto tautomeric conversion of phenylpyruvate (ketonase reaction) was assessed at room temperature by monitoring the decrease in absorbance at 288 nm on a dual path Shimadzu 2100 UV spectrophotometer (Shimadzu, Kyoto, Japan) according to Taylor et al.^55^ with minor modifications. Briefly, the 0.72 ml reaction mixture consisted of the enzyme at a final concentration of 0.4 µg/ml and the substrate in a 50 mM sodium phosphate buffer (pH = 6.5). The phenylpyruvate substrate was dissolved fresh in absolute ethanol to yield a final concentration of 100 µM. The reaction was monitored for 75 s at room temperature. A recombinant human MIF (from ATGen Co. Ltd., Seoul, Republic of Korea) was used as the enzyme source. Inhibitors were dissolved fresh in ethanol or DMSO. Ethanol or DMSO did not affect the enzyme reaction at the amounts applied. The compounds were diluted to achieve final concentrations of 200, 100, 50, 20, 5, 1, and 0.5 µM in the reaction mixture. All data presented have been derived from consonant measurements repeated at least three times. As reference inhibitor caffeic acid (ketonase and enolase IC_50_: 0.5 and 2.0 µM respectively) was used^28^. The areas under the curves obtained with and without the inhibitors were used to compare the conversion rates of the substrate. The IC_50_ value – the concentration of the given compound required to achieve 50% inhibition^56^ –was obtained by a nonlinear curve fitting (Sigma Plot 2000, SPSS Inc., Chicago, IL, USA) on the data obtained with the different concentrations of the inhibitor using the equation: f = min+(max − min)/(1+(*x*/EC50)nHill).

### Molecular modelling

Molecular modelling was applied as a means of gathering information on the structure’s binding mode. Glide docking was conducted on various MIF-ligand crystallographic structures in Extra Precision mode without constraints. Dockings with core restrictions using two types of docking output structures were examined to assess the favoured binding mode. Covalent docking was used to visualise the hypothesised adduct formation with Pro1. Molecular Dynamics simulations were used to assess the stability of the reversible complexes and explore the nature of the binding site interactions.

The crystallographic structure of MIF in complex with an inhibitor was obtained from Protein Data Bank (PDB 6B1K). All calculations were carried out with the modules of Schrödinger Suites 2019–2 (Schrödinger, LLC, New York, NY) in Maestro. The protein was prepared by adding hydrogens and missing side chains. Water molecules, sulphate ions and glycerol molecules were removed, as well as ligands in the active sites formed by chains A–B and B–C. Hydrogen bonds were optimised at pH = 7.4, followed by Impref minimisation using OPLS3e force field. The 3D structures of the ligands were determined by LigPrep at pH = 7.4 using OPLS3e force field. The grid box for docking was centred on the original ligand between chains A and C with automatic ligand size. Initial Glide docking experiments were run without any pharmacological constraints. Type I constrained docking was run with a tolerance of 0.1 Å, while in Type II constrained docking, the tolerance was set to 2 Å. Docking scores and MM/GBSA calculation results of reversible complexes in kcal/mol are summarised in Table 2. The model was validated by docking 3-(3,4-dihydroxyphenyl)-7-hydroxy-4*H*-chromen-4-one (RCSB ligand ID 47X, co-crystallized in MIF PDB entry 3L5R) with extra precision and without using constraints. Four viable poses were found, all of them similar to the binding mode observed in 3L5R, with docking scores ranging between −9.975 and −10.533 kcal/mol. Ligand RMSD values ranging from 0.7428 to 1.6388 Å for all heavy atoms were calculated, while values between 0.3643 and 0.7292 Å were calculated for the 7-hydroxy-4*H*-chromen-4-one core residing in the solvent inaccessible binding pocket of MIF. RMSD values for the ligand were calculated after binding site alignment considering residues within 5 Å range of the ligand, and 3L5R was used as the reference structure in its native form.

**Table 2.** Glide XP docking scores and CovDock affinity scores of the compounds using type I and type II docking constraints.

	Reversible				Covalent			
	Type I		Type II		Type I		Type II	
	docking score (XP)	MM/GBSA dG Bind	docking score (XP)	MM/GBSA dG Bind	cdock affinity	MM/GBSA dG Bind	cdock affinity	MM/GBSA dG Bind
**1**	−8.499	−54.71	−8.519	−57.99	−8.693	−61.64	−6.249	−37.79
**2**	−8.132	−50.83	−7.819	−55.61	−8.233	−34.65	−6.685	−36.06
**3**	–	–	−6.134	−28.08	−7.312	−24.51	−6.981	36.11
**4**	−8.213	−52.36	−7.034	−42.78	−8.188	−35.80	−6.140	−47.58
**5**	−8.210	−53.75	−4.216	−46.44	−8.141	−37.61	−5.607	−24.69
**6**	−8.135	−52.37	−7.225	−38.32	−8.269	−45.84	−6.819	−27.79
**7**	−8.309	−55.48	−8.360	−51.57	−8.045	−50.49	−7.748	6.62
**8**	−8.193	−51.5	−6.560	−39.76	−8.046	−36.68	−6.685	−43.60
**9**	−8.052	−53.51	−5.437	−36.13	−8.183	−37.85	−6.176	−34.18
**10**	−8.204	−50.87	−6.788	−53,12	−8.626	−41.35	−6.855	−34.79
**11**	−8.236	−57.97	−7.176	−20.02	−7.897	−42.24	−7.804	46.15
**12**	−7.926	−52.70	−6.785	−44.62	−8.186	−40.35	−6.525	−48.47
**13**	−8.278	−57.06	−7.075	−42.91	−8.024	−52.93	−7.111	−32.16
**14**	−8.209	−51.36	–	–	−8.301	−40.16	−5.758	−29.96
**15**	−8.221	−52.12	−4.795	−2.46	−8.369	−37.02	−6.363	−47.81
**16**	−8.281	−51.63	−6.784	−41.76	−7.457	−45.26	−6.289	−43.93
**17**	−8.369	−54.02	–	–	−8.464	−45.79	−5.367	−44.99
**18**	−8.143	−50.91	−6.521	−41.23	−8.197	−46.33	−6.542	−41.71
**19**	−7.935	−53.14	−7.953	−57.54	−7.890	−41.16	−6.452	−44.63
**20**	−8.063	−54.70	−7.505	−54.38	−8.308	−34.01	−6.735	−32.73
**21**	−8.019	−51.92	−7.732	−54.85	−8.000	−28.22	−7.221	−34.68
**22**	−7.929	−54.38	−6.722	−49.94	−7.823	−39.63	−6.360	−18.93
**23**	−8.200	−50.17	−6.152	−36.16	−8.344	−39.62	−6.480	−35.48
**24**	−8.227	−55.40	−7.714	−60.28	−8.504	−42.35	−7.072	−33.41
**25**	−8.093	−50.90	−6.606	−53.61	−8.256	−41.14	−7.077	−35.23
**26**	−8.206	−50.79	−6.501	−54.63	−8.601	−40.48	−6.242	−32.12
**27**	−8.425	−56.44	−6.836	−56.88	−8.533	−48.12	−6.750	−34.59
**28**	−8.289	−58.79	−7.046	−44.12	−8.837	−48.69	−6.608	−33.40
**29**	−8.196	−58.01	−6.252	−43.46	−8.228	−49.88	−7.206	−28.65
**30**	−8.315	−58.44	−4.182	−47.12	−8.667	−50.57	−5.632	−36.95
**31**	−8.132	−54.03	−4.606	−41.29	−8.560	−51.83	−6.300	−34.00
**32**	−8.500	−61.06	−6.732	−15.06	−8.626	−45.56	−6.887	9.84
**33**	−8.341	−57.21	−6.865	−48.23	−7.404	−50.21	−6.634	−39.94
**34**	−8.195	−54.00	−8.28	−43.08	−8.134	−44.49	−7.360	−27.69
**35**	–	–	–	–	–	–	–	–
**36**	−7.700	−37.08	−6.352	−48.78	−7.495	−25.07	−6.765	−27.60
**37**	–	–	–	–	–5.623	–26.39	–6.764	–32.26
**38**	–	–	–	–	–5.975	12.70	–	–

Calculated binding energies are provided for both, the reversible and covalent MIF-ligand complexes.

Covalent dockings were run in CovDock within Maestro. The following custom covalent mechanism protocol was created for this purpose:

LIGAND_SMARTS_PATTERN 1,[C] = [C]-[C] = [O]

RECEPTOR_SMARTS_PATTERN 2,[C]-[N;H1,-1]

CUSTOM_CHEMISTRY ("<1>",("charge",0,1))

CUSTOM_CHEMISTRY ("<1>|<2>",("bond",1,(1,2)))

CUSTOM_CHEMISTRY ("<2> =[C]-[C] = [O]",("bond",1,(1,2)))

Pro1A is present in the conjugate acid form, and was manually deprotonated before initiating covalent dockings. The same core constraint settings were applied as to the Glide docking experiments. The tolerance was set to 2 Å in both instances. The pose prediction docking mode was used. Docking scores and MM/GBSA calculation results of covalent complexes in kcal/mol are summarised in Table 2.

Molecular dynamics simulations ranging between 1.2 and 175 ns were applied to a selected group of the compounds without using constraints. All processes were initiated using Desmond in Maestro. Output structures of a docking experiment with Type I core constraints were used as starting points. SPC solvent model was used and box boundaries were adjusted to a distance of 10 Å from the protein–ligand complexes in each axis. Net positive charge of the protein was neutralised by chloride ions. 0.15 M NaCl was added to the systems in each instance. Simulations were carried out using NPT conditions, at a constant temperature of 300 K and pressure of 1 atm, with Nosé–Hoover chain thermostat (1 ps relaxation time) and MTK barostat (2 ps relaxation time) methods. The cut-off radius for coulombic interactions was set to 9 Å. The systems were relaxed before simulation using the default relaxation process of Desmond^57^. The stability of (**24**) in the active site was explored through a 175 ns simulation with 25 ps recording intervals, and the Thermal MMGBSA script available in Schrödinger was used to obtain multiple binding free energy values for the complex, sampling every 100th frame of the simulation. The 1.2 ns simulation of (**24**) was also conducted with TIP3P water model to explore the nature of interactions on the ps scale in a different MD setup.

### Cell culture and treatments

In cell culture experiments, we used RAW264.7 mouse monocyte/macrophage cell line (ECACC, Salisbury, UK; passage number: 8–15) and RAW-Blue™ cells (Invivogen, Toulouse France, passage number: 10–14). RAW264.7 and RAW-Blue™ cells were cultured in 5% CO_2_ at 37 °C in endotoxin-tested Dulbecco’s Modified Eagle’s Medium (high glucose, 4.5 g/L, 2 mM l-glutamine; Corning, Corning Incorporated Costar, Corning, NY) supplemented with 10% FBS. For culturing RAW-Blue™ cells, we also added 100 µg/ml normocin and 200 µg/ml zeocin to the medium. The day before the experiments, cells were plated onto 24- or 96-well plates and cultured overnight. Then medium was replaced to fresh one and cells were induced by 0.1 µg/ml or 1 µg/ml lipopolysaccharide (LPS; *E. coli*, 0127:B8; Sigma-Aldrich, Budapest, Hungary). The freshly synthesised tetralone derivatives were dissolved in DMSO and applied in 20 µM concentration as a pre-treatment, added 30 min before LPS induction. To exclude the effects of vehicle, both CTRL and LPS-treated cells received the same amount of DMSO.

### ROS and nitrite production

To measure the amount of reactive oxygen species we seeded RAW264.7 cells onto 96-well plates in a density of 10^5^cells/well. Cells were treated with the tetralones as a pre-treatment and they were induced with 1 µg/ml LPS for 24 h. Then 2 µM dihydrorhodamine123 (DHR123; Life Technologies, Carlsbad, CA) fluorescent dye was added and incubated for an additional 2 h. Fluorescent intensity of the dye (excitation 490 nm/emission 510–570 nm) was measured with Glomax Multi Detection System (Promega^®^, Madison, WI, USA).

For nitrite measurements, we applied the same culturing conditions, treatments, and equipment as described above. After 24 h of incubation, 50 µl medium was removed and added to equal amount of Griess reagent (Sigma-Aldrich, St. Louis, MO, USA) in 96-well plate. The optical density was measured at 550 nm wavelength.

### NF-κB activation

The detection of NF-κB activation was accomplished by using RAW-Blue™ cells. RAW-Blue™ cells are genetically modified RAW264.7 macrophages, which are able to secrete embryonic alkaline phosphatase (EAP) upon LPS induction and following NF-κB activation. The levels of EAP can be examined using QUANTI-Blue™ detection medium.

RAW-Blue™ cells were seeded into 96-well plate at a density of 10^5^cells/well. Cells were treated with tetralones (pre-treatment, 20 µM) and with 1 µg/ml LPS. 24 h after LPS treatment 20 µl medium was removed and incubated with 100 µl QUANTI-Blue™ detection medium (Invivogen; Toulouse, France) in 96-well plates. The optical density was measured at 600 nm.

### Cytokine production

For cytokine concentration measurements, RAW264.7 cells were cultured in 24-well plates at a density of 5*10^5 ^cells/well and treated with tetralones (pre-treatment, 20 µM) and with 1 µg/ml LPS for 24 h. TNF-α, IL-6 and CCL-2 levels were determined from the culturing media by using Ready-Set-Go ELISA kits (eBioscience, San Diego, CA, USA) for TNF-α and IL-6 detection and mouse CCL-2 uncoated ELISA-kit (Invitrogen, Vienna, Austria) for CCL-2 determination. ELISA-kits were applied as provided by the protocol of the manufacturer, the optical density was measured at 450 nm.

### Mouse thermometry studies

Thermophysiological experiments were performed in 24 C56BL/6 adult male mice. Experimental procedures were approved by the Animal Research Review Committee of the University of Pécs, Medical School (Permit number: BA02/2000–6/2018). At the time of the experiments, the mice weighed 28 ± 4 g. Animals were housed in temperature-controlled rooms on a 12/12 h light/dark cycle. Standard rodent chow and tap water were available *ad libitum*. All experimental procedures were carried out according to the European Communities Council Directive of 2010/63/EU under protocols approved by the Institutional Animal Use and Care Committee of the University of Pécs. Mice were anaesthetised with an intraperitoneal (i.p.) injection of ketamine-xylazine (81.7 and 9.3 mg/kg, respectively) and received antibiotic prophylaxis intramuscularly (gentamycin, 6 mg/kg). During the surgery, mice were kept on a heating pad (PECO Services Ltd., Brough, UK), and then to prevent postsurgical hypothermia, the animals were allowed to recover from anaesthesia in a temperature-controlled chamber (model MIDI F230S; PL Maschine Ltd., Tarnok, Hungary) set to 31 °C. Each mouse was implanted intraperitoneally with a miniature biotelemetry transmitter (G2 E-Mitter series; Starr Life Sciences Corp., Oakmont, PA) to record abdominal temperature (Tab) and general locomotor activity. The latter has been shown to play an important thermoregulatory role in mice^58–60^. The transmitter was inserted into the abdominal cavity through a small midline incision and was fixed to the left side of the abdominal wall with suture. The surgical wound was closed and the mice were returned to their home cages. The mice were allowed a 2–3 weeks post‐surgical recovery period before an experiment.

Telemetry receivers (model ER-4000; Starr Life Sciences Corp., Oakmont, PA, USA) were positioned in a temperature-controlled room, and the home cages of mice were placed on top of the receivers, as described earlier^59^. The ambient temperature of the room was set to 26 °C, which is near the lower end of the thermoneutral zone for mice^61^. On the day of an experiment, compound (**24**) at 1.6 mg/kg or its vehicle (10% DMSO in saline) was administered intraperitoneally in bolus (3.3 ml/kg). Thirty minutes later, either LPS (5.0 mg/kg) from *Escherichia coli* 0111:B4 (Sigma-Aldrich, St. Louis, MO) or saline was intraperitoneally injected to the mice.

Thermoregulatory responses were compared by two-way ANOVA followed by Fisher LSD *post hoc* test, as appropriate, using Sigma Plot version 11.0 (Systat Software, San Jose, CA, USA) with the level of significance set at *p* < 0.05. The data are reported as means ± SEM.

## Results

### Tautomerase assay

First the inhibitory effect on the ketonase reaction is discussed. As regards the structure–activity relationship, the size of the cyclic ketone ring has an impact on the inhibitory effect (see Table 1). From the phenyl substituted compounds, the five-membered (**1**) and the seven-membered (**3**) showed almost identical inhibitory effect (IC_50_=82.8 and 84.0 µM, respectively), while the six-membered (**2**) exerted a lower activity (IC_50_=127 µM). The substituted derivatives of (**2**) containing both homoaromatic and heteroaromatic side chain were examined.

Summarising the results of the measurements studying the possible inhibitors of ketonase reaction, the best inhibitors are the compounds with a nonpolar aromatic side chain, such as the methyl derivative (**4**), the *ortho,para*-dichloro compound (**13**) and the polar 2-pyridyl derivative (**24**). Analysis of the structure–activity relationship is presented later on in more detail.

The inhibition of the enolase activity was also investigated. In the group of the compounds with a homocyclic skeleton, some good inhibitors can be found, such as five-membered phenyl derivative (**1**) (IC_50_=20.2 µM). Compounds with a heteroaryl side chain produce the highest bioactivity. From the five-membered derivatives the indolyl- (**23**, IC_50_=2.89 µM), the furyl derivative (**19**, IC_50_=25.4 µM) and the *N*-metylpyrrolyl compound (**22**, IC_50_=27.8 µM) are the best inhibitors. As for the six-membered ones, two pyridyl derivatives (**24** and **25**, IC_50_=28.6 µM for both) exerted the best activity, while the 4-pyridyl derivative (**26**) proved to be a weaker enolase inhibitor. Comparing the enolase and ketonase activity and focussing on the selectivity, some of our compounds acted as good ketonase inhibitors having high selectivity, such as (**4**) (IC_50_enolase/IC_50_ketonase = 13.9), (**13**) (IC_50_enolase/IC_50_ketonase =18), (**26**) (IC_50_enolase/IC_50_ketonase =10), while the substance (**23**) (IC_50_ketonase/IC50enolase =20) behaved as a strong and selective enolase inhibitor.

### Molecular modelling

#### Docking experiments

The majority of MIF inhibitors target the active site where enzymatic reactions take place. The protein is known to form homotrimers, in which binding sites are formed by each of two adjacent chains. As represented in the Protein Data Bank entry 3IJJ, 4-hydroxyphenylpyruvate contacts with Pro1, Lys32 and Ile64 of monomer A, and Tyr95 and Asn97 of monomer C. Available structures of MIF in complex with various inhibitors reveal the importance of these residues in ligand binding^62^. Like the series of compounds presented in this paper, many MIF inhibitors contain more than one aromatic ring^63^, suggesting the extensive involvement of π-interactions.

#### Reversible docking

**Figure 2.**
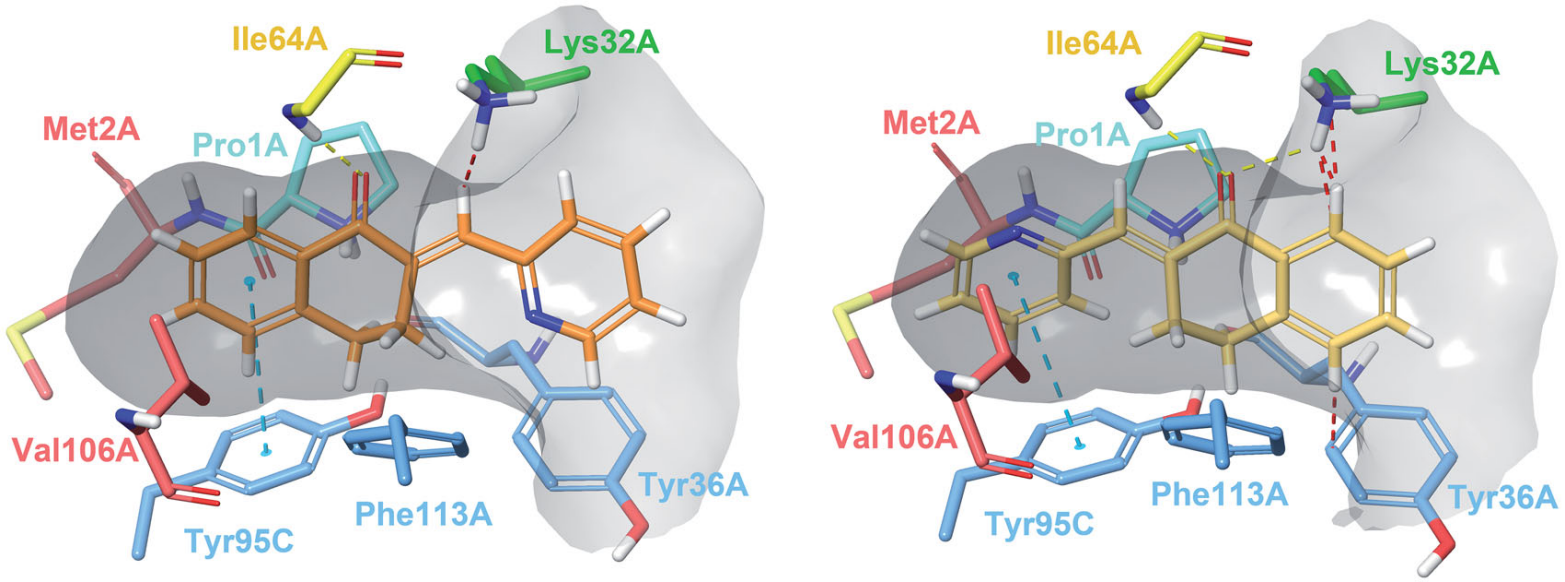
Type I (left) and Type II (right) binding modes of tetralones in the active site of MIF, identified through reversible docking experiments. Carbons of (**24**) are coloured orange (Type I) and yellow (Type II). Residues are coloured according to their type of interaction with the ligand: yellow residues form H-bonds, blue residues form π–π stacking and green form cation-π interactions, red residues are hydrophobic, and the catalytic Pro1 is coloured cyan. The grey area is the inner surface of the binding site. Red dashed lines indicate steric clashes.

Docking experiments revealed two possible types of binding mode (Figure 2). In Type I complexes, the A and B rings occupy the binding pocket. The ketone oxygen forms a hydrogen bond with the peptide NH of Ile64A. The aromatic ring interacts with Tyr95C through π–π stacking. The catalytic Pro1A and Lys32A are in the proximity of the methylene bridge. The aryl or heteroaryl group bonds with Lys32A, Tyr36A and Phe113A through hydrophobic and π-interactions. Such binding mode can be observed in the PDB entry 3L5R of a 3-phenylchromen-4-one compound^64^. In type II binding mode, where the ligand is horizontally flipped, the C-ring interacts with Tyr95C, the ketone oxygen with Ile64A and Lys32A, and the A ring with Tyr36A. A similar binding mode of a structurally related covalent inhibitor can be observed in the PDB entry 4Z1U^65^. After visual analysis and consideration of docking scores, we propose Type I binding mode to be more favourable.

#### Covalent adduct formation

In the reversible docking models, the position of the electrophilic methylene bridge relative to Pro1A hints at the possibility of a covalent mechanism. Such catalytic proline residues reportedly possess the ability to mediate enzymatic conversions through covalent catalysis^66^. NAPQI has been shown to bind covalently to Pro1, while exhibiting non-covalent inhibitory activity^67^. The covalent mechanism of a series of MIF inhibitors has also been verified by X-ray crystallography and enzymatic assays^65^, where both covalent and non-covalent tautomerase inhibitory activity have been attributed to “compound 12”, visible in PDB entry 4Z1U^65^.

**Figure 3.**
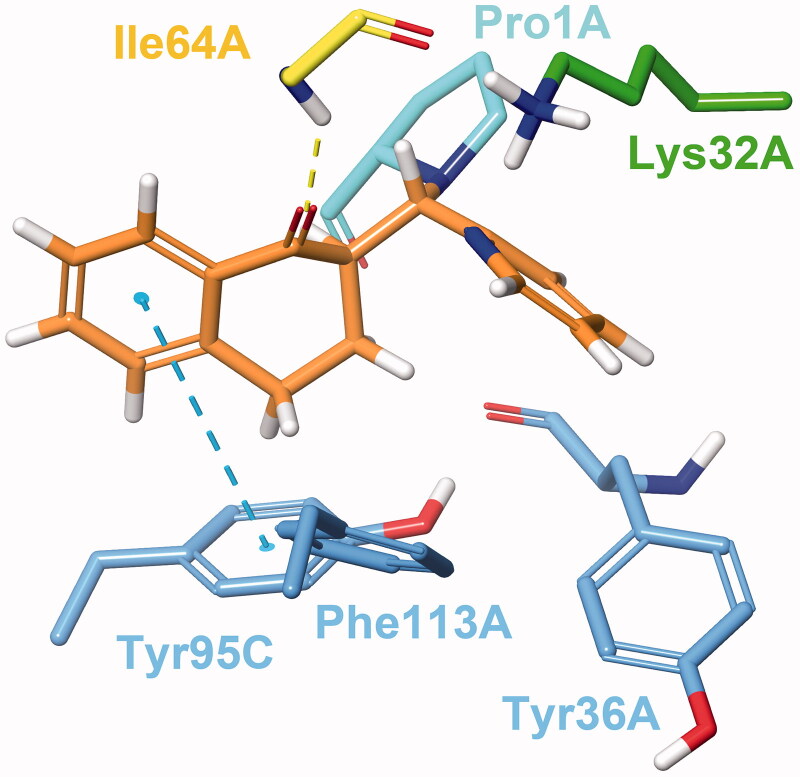
Compound **24** covalently bound to Pro1A of MIF in Type I binding mode. The H-bond with Ile64A is represented by the yellow, and the π–π stacking with Tyr95C by the blue dashed line. The docking output structure was minimised to avoid clashes due to the rigid receptor model. Active site residues involved in reversible binding are shown. Some atoms are hidden for clearer view of the covalent adduct.

Both Type I and Type II constraints were used for covalent docking to assess whether any of the two determined binding poses would be favourable for a covalent reaction to occur between Pro1A and the methylene carbon. Covalent adduct formation was successful for most molecules, with the exception of (**35**) and (**38**) due to steric clashes. Covalent docking affinity and MM/GBSA scores indicate that the reaction is more likely to happen in Type I binding mode (Table 2). Even though an increased number of steric clashes with Asn97C, Tyr95C and Pro1A are visible, minimisation of the output structure results in a reasonable covalent adduct (Figure 3).

#### Molecular dynamics

We conducted MD simulations to reveal binding characteristics obscured by using a rigid protein structure for docking. The H-bond between Ile64A and the ketone oxygen is a crucial contact. The π–π stacking between the A ring and Tyr95C is a T-type stacking interaction. Weak hydrophobic contacts with Val106A and Met2A are present. The arylmethylene substituent is exposed to the solvent (see ligand RMSF in Supplementary material, Figure S2) and forms a sandwich-type π–π stacking with Tyr36A. Rotation of the C-ring is hindered by constant interactions with Lys32A, Tyr36A and Phe113A (Supplementary material, Figure S3). A steric clash may occur for 3-substituted phenylmethylene-tetralones such as the inactive analogue (**9**). The protonated amino group of Lys32A is capable of forming a cation-π bond with the C-ring, especially of heteroaryl derivatives (**22–26**). The interaction is less pronounced for phenyl compounds, such as (**4**) and (**13**), and virtually absent from models of (**9**). All three interactions can be present concurrently. A water molecule can also be involved in ligand binding, where the catalytic Pro1A and the adjacent Tyr36A interacts with the ligand through a water bridge. The hydrogen of the water molecule occasionally engages in close contact with the methylene carbon or interacts with heteroatoms and halogen substituents of the C ring.

**Figure 4.**
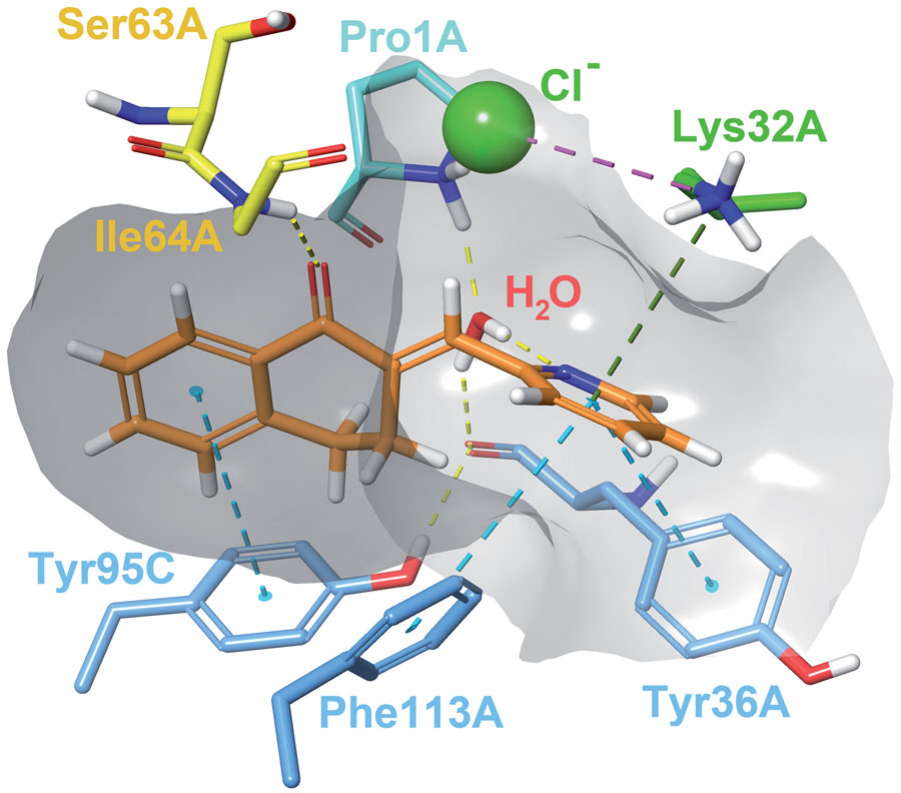
Interactions between the active site residues and compound **24** as it maintains its binding conformation. Both an active site-bound water molecule and a chloride ion are visible. Hydrogen bonds are represented by yellow, π–π stackings by blue, cation-π interactions by green and ionic bonds by purple dashed lines. The grey area represents the inner surface of the binding site. Some atoms of active site residues are not shown for clearer view on interactions.

The importance of the interaction is most pronounced in the case of (**24**), where the 2-pyridyl nitrogen is adequately positioned to maintain a constant water bridge interaction (Figure 4). Consequently, a stable complex forms between (**24**) and MIF, where no major conformational change is observed for 65 ns. The presence of interactions in percentage of frames are: Ile64A_(H-bond)_ = 65%; Tyr95C_(π–π)_ = 64%, Lys32A_(cation–π)_ = 69%, Pro1A_(water bridge)_ = 91%, Tyr36A_(water bridge)_ = 83%. Additionally, π–π interactions with Tyr36A and Phe113A were observed in 18% of the frames for both residues. The most prominent weak hydrophobic contacts are formed with Met2A, Tyr36A, Val106A and Phe113A and are present throughout the entirety of the simulation. The averaged free binding energy was calculated at −59.32 kcal/mol (max= −51.19, min= −68.75; SD = 3.40) for this phase. Minor protein and ligand movements at 65 ns result in (**24**) assuming a minimally altered, but stable position until 138.79 ns. In this phase of roughly 73 ns in length, H-bond interaction with Ile64A diminish to 21% as it forms weak hydrophobic contacts instead, and π–π interaction with Tyr95C falls to 2%. The water bridge interaction becomes even more pronounced with 94% and 90% presence rates for Pro1A and Tyr36A. Lys32A and Phe113A continue to interact with the π –electron system of the C ring (60% and 32%). Free binding energies for this phase were calculated at an average of −54.82 kcal/mol (max= −46.95, min=-63.18; SD = 3.88). At 138.79 ns Trp108A disrupts binding as it intrudes the active site, which results in cessation of most H-bond and π interactions, and the distancing of the methylene carbon from Pro1A as well. As a result, (**24**) undergoes a major positional shift, and only π–π stacking interactions of the C-ring with Phe113A and Trp108A are visible with 41% and 27% frequencies. Weak hydrophobic contacts with Phe49C and Tyr95C also develop. The complex remains unchanged until the end point of the 175 ns simulation (see Supplementary material Figures S4–S7 for RMSD plot and active site residue contributions).

In simulation of (**24**) using TIP3P water model, the phenolic oxygen of Tyr95C acts as the H-acceptor instead of Tyr36A; such model describing an enolate intermediate was published previously^68^. We have also identified a potential chloride ion binding motif at the active site. Chloride binding in MIF may be a remnant of a lost dehalogenase function. Such enzymatic activity is exhibited by structurally related bacterial tautomerases CaaD and *cis*-CaaD, of which the former also possesses a phenylpyruvate tautomerase activity similar to that of MIF^6^.

#### Structure–activity relationship

Both aromatic rings interact with active site residues. The ideal size for the B ring is either 5 or 6 atoms without any substituents; larger ring systems are less favourable. The 1-tetralone ring is preferred over 4-chromanones, and thiochromanone analogues have even weaker effect. The *ortho*-substitution of the C ring, as well as switching an *ortho*-carbon to nitrogen, is favourable for a water bridge interaction with Pro1A to occur. The heterocyclic nitrogen of (**24**) and, to a lesser extent, the 2-chloro substituent of (**13**), are able to bind a water molecule. For the 3-pyridyl and 4-pyridyl analogues (**25**) and (**26**), or the 3-bromo substituted (**9**), no persistent water bridge interaction is observed. Certain *para*-substituents are partially solvated, or can interact with the phenolic hydroxyl of Tyr36A. Some may also stabilise the molecule and prevent changes in binding pose. *Meta*-substituents seem to prevent tighter binding, possibly due to steric clashes with proximal residues. Changing in the phenyl group *meta*- or *para*-carbon to nitrogen, also enhances inhibitory activity, although to a lesser extent compared to the 2-pyridyl analogue. Changing the phenyl ring to a 5-membered heterocycle mostly increases activity, although not for unsubstituted pyrrolyl derivative (**21**), that shows somewhat weaker effect compared to the phenyl analogue. Electron rich derivatives such as (**23**) are more likely to interact with the active site lysines, while electron-deficient rings are less likely to do so.

### Macrophage activation

#### Tetralone derivatives reduce ROS and NO production in LPS-induced macrophages

**Figure 5.**
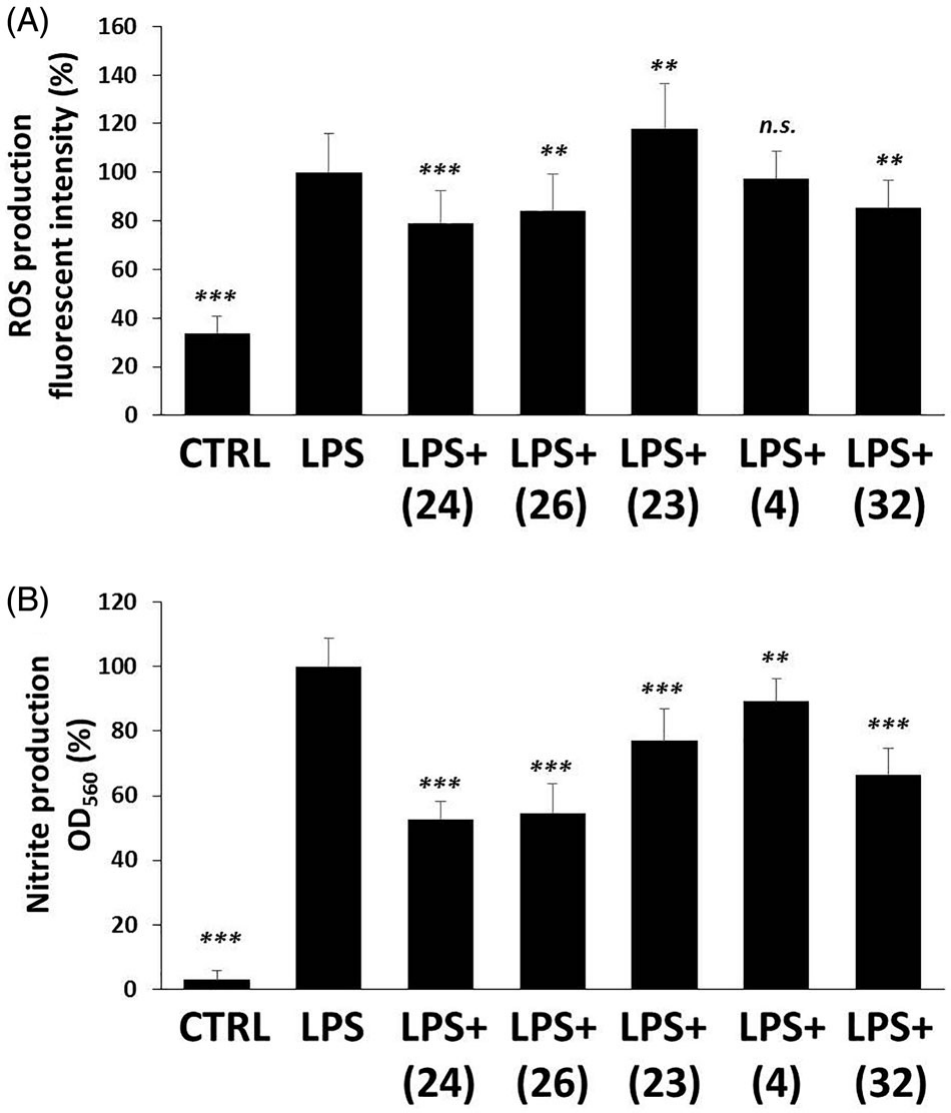
Tetralone derivatives reduce ROS and nitrite production. RAW264.7 cells were pre-treated with tetralones for 30 min and they were induced by 1 μg/ml LPS for 24 h. (A) ROS production was measured by using 2 μM dihydrorhodamine 123 fluorescent dye (fluorescent intensity was measured at 490 nm [excitation]/510–570 nm [emission] wavelengths). (B) Nitrite production was evaluated by applying Griess reagent (optical density was measured at 550 nm). Data are presented as means ± SD in percentage of LPS-treated group; *n* = 3 × 6 (pooled data of 3 independent experiments with 6 parallels); Student’s *t* test, *p* values < 0.05 were considered significant. * *p* < 0.05; ** *p* < 0.01; *** *p* < 0.001; n.s. = non-significant.

MIF has been involved for long in the pathomechanism of inflammation including macrophage activation. One of the most important signs of inflammatory macrophage induction is the production of reactive oxygen and nitrogen species, like superoxide (O^•^_2_^−^) and nitrogen monoxide (NO^•^)^69^. Therefore, we measured ROS and nitrite (NO rapidly oxidises to NO_2_^−^ in the medium of cells) production of LPS-induced and tetralone derivatives-treated RAW264.7 macrophage cells. 24 h after LPS treatment we detected a significant, approximately ∼3-fold increase in ROS production compared to the basal level of vehicle-treated control cells (Figure 5(A)). Three out of the five selected compounds slightly inhibited ROS production. To be more precise, we detected at about ∼20% decrease in ROS concentrations of (**24**)-, (**26**)- and (**32**)-treated, LPS-activated cells. In contrast to these (**4**) failed to modify ROS level and surprisingly, (**23**) induced a slight increase in it. Nitrite concentrations were found to be considerably increased (∼20-fold) by LPS treatment (Figure 5(B)), however in the media of (**24**)-, (**26**)- and (**32**)-treated active macrophages, we detected markedly lower nitrite concentrations (∼50%). Interestingly, compounds (**4**) and (**23**) could also inhibit nitrite production, but to a lower extent.

#### Majority of tetralone derivatives diminish NF-κB activation in macrophages

**Figure 6.**
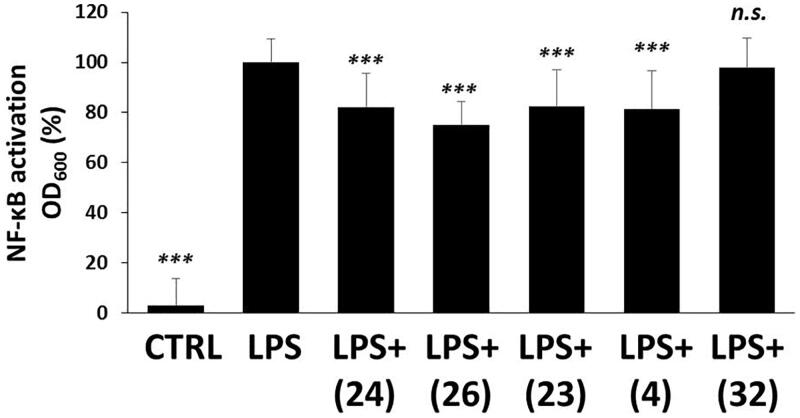
The effect of tetralone derivatives on NF-κB production. NF-κB activation in macrophages was evaluated by using RAW-Blue™ cells. Macrophages were pre-treated with tetralones for 30 min and they were induced by 1 μg/ml LPS for 24 h. The activity of secreted embryonic alkaline phosphatase was measured by using QUANTI-Blue™ detection medium. The optical density was measured at 600 nm. Data are presented as means ± SD in percentage of LPS-treated group; *n* = 3 × 6 (pooled data of three independent experiments with six parallels); Student’s *t* test, *p* values <0.05 were considered significant. ***p < 0.001; n.s.=non-significant.

NF-κB is an inflammatory transcription factor, which can be activated by PAMPs, like LPS via TLR4^70^ or even by oxidative stress^71^ and induces proinflammatory protein expression. LPS induced a dramatic increase (∼40-fold) in the transcriptional activity of NF-κB compared to DMSO-control (Figure 6). We found slight, but statistically significant inhibitory effect in the case of (**24**), (**26**), (**23**) and (**4**) treatments, in relation to the LPS-activated cells. Compound (**32**) could not show any reducing effect on NF-κB activation.

#### Tetralone derivatives modulate cytokine and chemokine production in RAW264.7 cells

**Figure 7.**
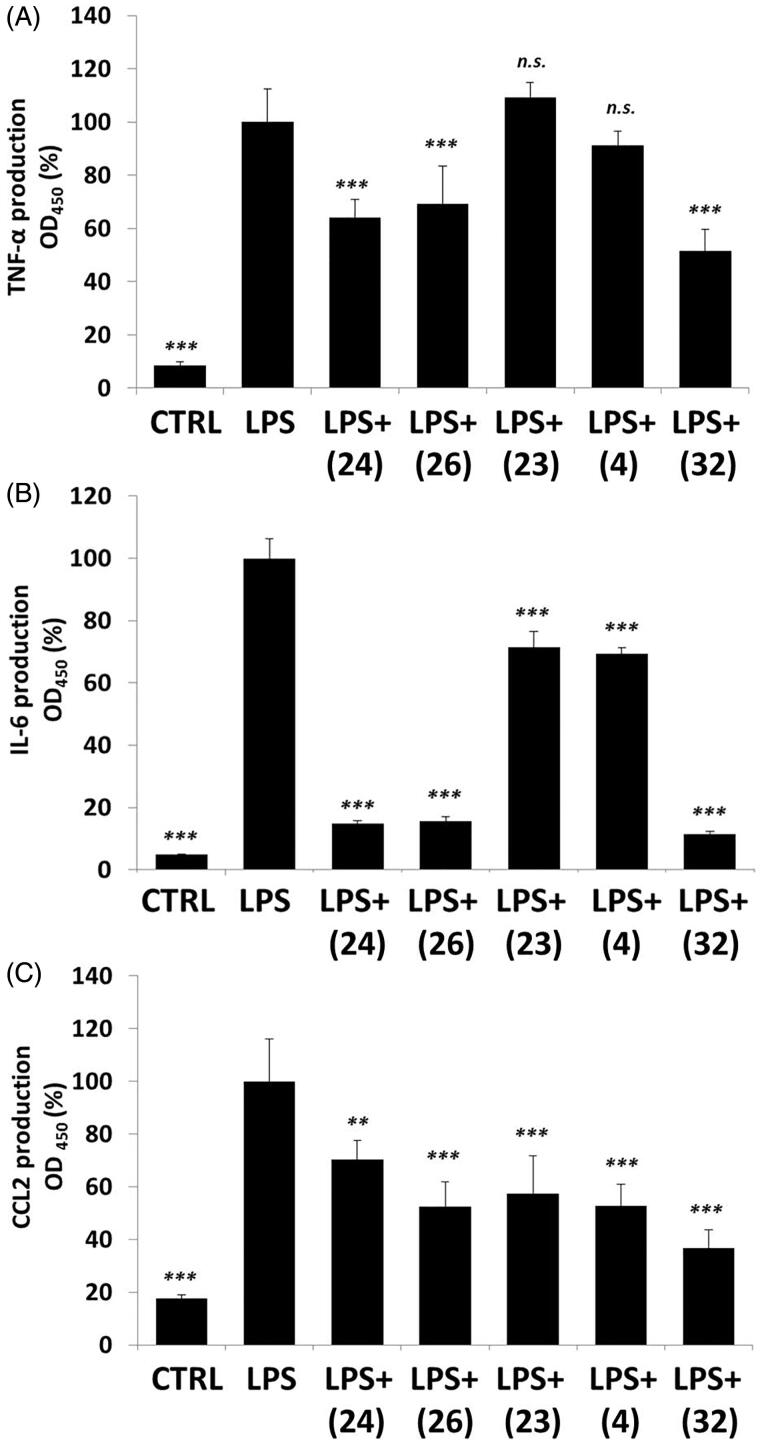
Cytokine production of activated macrophages: (A) TNF-α; (B) IL-6; (C) CCL-2. LPS-activated RAW264.7 cells were pre-treated with tetralones for 30 min and they were induced by 1 μg/ml LPS for 24 h. Cytokine concentrations were measured with ELISA-kits, optical density was measured at 450 nm. Data are presented as means ± SD in percentage of LPS-treated group; *n* = 6; Student’s *t* test; *p* values < 0.05 were considered significant. **p < 0.01; ***p < 0.001; n.s. = non-significant.

NF-κB participates in the protein expression of many inflammatory cytokines, like TNF-α, IL-6^72^ and chemokines like, CCL2^73^. Levels of TNF-α, IL-6 and CCL2 were measured in the media of activated macrophages upon tetralone treatments. LPS induced a powerful increase (∼5–20-fold) in the concentrations of all investigated cytokines and chemokines (Figure 7). (**24**), (**26**) and (**32**) reduced TNF-α production, but (**23**) and (**32**) failed to do so (Figure 7(A)). In case of IL-6 all of the five selected tetralone compounds could diminish IL-6 production, but (**24**), (**26**) and (**32**) had the most dominant effect (Figure 7(B)). CCL2 levels were also negatively modified by all of the investigated tetralones, which showed comparable inhibitory efficacy (Figure 7(C)).

### Thermophysiology

**Figure 8.**
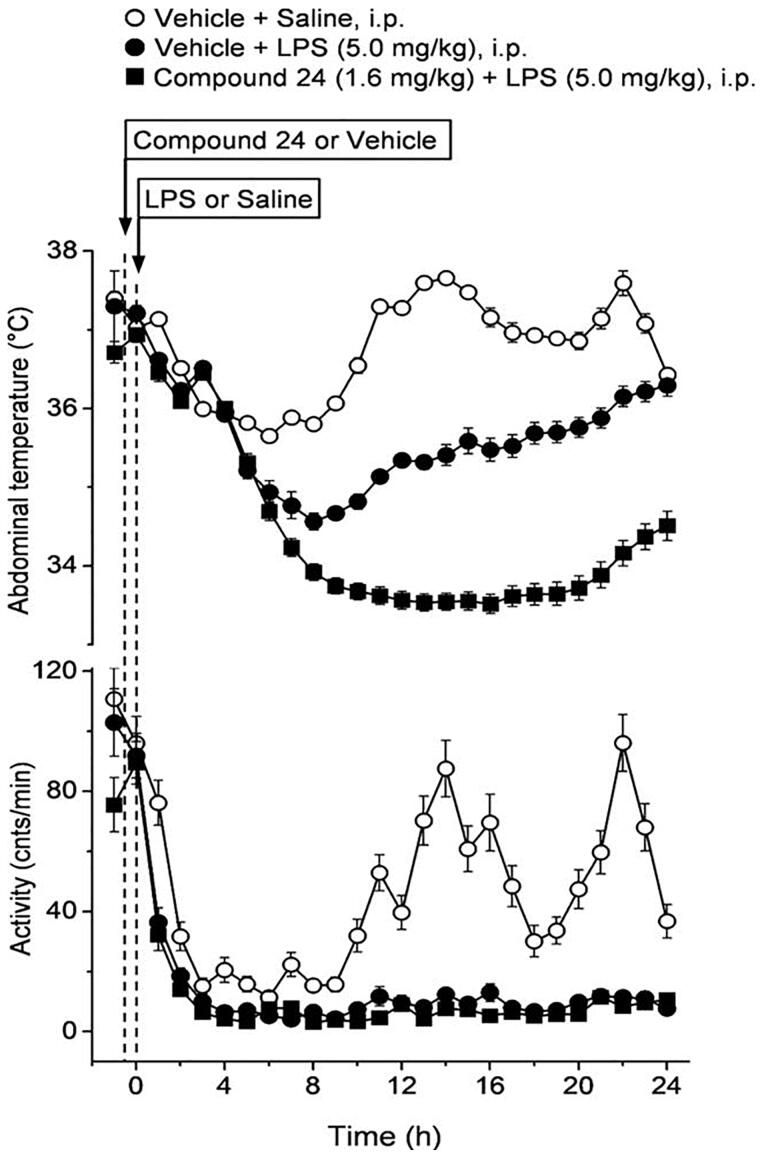
The effect of compound 24 on thermoregulation and locomotor activity in mice. The effects of compound 24 (or vehicle) pre-treatment at −30 min on the abdominal temperature (top) and locomotor activity (bottom) of mice treated with lipopolysaccharide (LPS) or saline at time zero (doses indicated). Pre-treatment with compound 24 significantly exaggerates the hypothermic response to LPS, while it does not further reduce the already near-zero level of locomotor activity in LPS-treated mice. The number of mice was 8 (*n* = 8) in each group. Data are presented as means ± SD.

The administration of compound (**24**) or its vehicle on its own did not cause any change in the abdominal temperature (Tab) and locomotor activity of the mice. On the contrary, the applied high dose (5.0 mg/kg i.p.) of LPS in a near subthermoneutral environment caused marked hypothermia and hypokinesis in vehicle-pre-treated mice (Figure 8), as expected^74^. Compared to saline, LPS caused a significant drop in Tab of vehicle-pre-treated mice from 8 to 22 h post-administration (*p* < 0.05), with the biggest intergroup difference of 2.3 °C at 13–14 h (*p* < 0.001). The LPS-induced hypothermia was brought about, at least in part, by suppressed locomotor activity, which was lower in LPS-treated than in saline-treated mice throughout the experiment and was significantly different between the groups at 11 h (*p* < 0.05) and from 13 to 22 h post-administration (*p* < 0.001) (Figure 8). Pre-treatment of the mice with compound (**24**) exaggerated and prolonged the LPS-induced decrease in Tab. In compound (**24**)-pre-treated mice the LPS hypothermia was significant from 7 to 24 h post-administration, compared to saline-treated mice, with the biggest intergroup difference of 4.1 °C at 14 h post-administration (*p* < 0.001). Importantly, the decrease of Tab in response to LPS was significantly more pronounced in mice pre-treated with compound (**24**) than in vehicle-pre-treated mice between 10 and 24 h post-LPS administration (*p* < 0.05). The LPS-induced decrease in locomotor activity could be also observed throughout the experiment after pre-treatment with compound (**24**), and did not differ from what was seen in vehicle-pre-treated mice. However, it should be noted that the locomotor activity was reduced to near-zero level in both LPS-treated groups, thus an exaggeration of the response (i.e. a further decrease) to compound (**24**) was hardly possible.

## Discussion

As regards the mechanism of action of the test compounds, we suppose, similarly to other authors, the importance of the *N*-terminal proline. The role of this basic amino acid in the mechanism of MIF was discussed by some researchers^75^. Mc Lean et al. studied hydroxyquinoline derivatives as efficient MIF inhibitors^76^. Applying crystallographic methods they were able to detect the covalent adduct formed in the reaction of Pro1 and an intermediate unsaturated ketone (“quinone methide”). Similar observations were made by Orita et al. also using hydroxyquinolines^25^.

Our test compounds, the cyclic α,β-unsaturated ketones are well known alkylators reacting with nucleophiles (amines, thiols) in the β-position in a Michael addition. They show a preferential affinity towards the thiols^77^. Thus, we suggest a Michael addition with Pro1 for our test compounds. This type of synthetic product has been described by Shankar et al. when reacting enones with piperidine^78^. The corroboration of the proposed mechanism is under progress.

Concerning potential anti-inflammatory activity of the tetralone analogues reported here, it is noteworthy that certain herbs used against inflammatory conditions are rich in tetralone substances^79^^,^^80^.

Next, we intended to give a picture of the biological efficacy of five preferred tetralone derivatives, namely (**4**), (**23**), (**24**), (**26**) and (**32**). These compounds were selected based on their excellent tautomerase inhibitor activity, i.e. they are potent MIF inhibitors (see Table 1). The most efficient compounds, namely (**24**): (IC_50_=5.63 µM for ketonase activity) or (**32**): (IC_50_=4.31 µM for ketonase activity) are even more powerful pharmacological inhibitors, than the well-known and widely accepted ISO-1 (14.41 µM)^23^. Since a strong association between tautomerase inhibition and attenuation of inflammatory macrophage activation exists^81^, and other MIF inhibitors were previously found to possess anti-inflammatory effects in LPS-induced RAW264.7 macrophages^82^, we decided to use this model in our investigations as well.

All of the chosen tetralone derivatives were found to have anti-inflammatory potencies in activated macrophages, but two of them (**24** and **26**) showed remarkably powerful effects. These two compounds inhibited ROS and NO production, NF-κB activation, and expression of the investigated cytokines (TNF-α, IL-6, CCL-2). It is important to note, that these molecules have similar structures; they are isomeric compounds where only the position of the nitrogen atom differs. Compound (**4**), however, with a quite similar methyl substituted structure was found to have only moderate effects compared to (**24**) and (**26**). The third best compound (**32**) had very similar effects on RONS and cytokine production, like (**24**) and (**26**), but surprisingly it could not inhibit NF-κB activation. Compound (**32**) is a methoxy substituted compound without nitrogen in the six membered C-ring and it has oxygen in its chromanone ring system compared to (**24**) and (**26**). Compound (**23**), the least effective tetralone in the investigated group also lacks a pyridyl ring, since it is an indolyl-compound. These findings might underline the importance of the pyridyl moiety in the biological activity of the investigated MIF inhibitors.

In thermophysiological experiments, we studied the effect of compound (**24**), a potent, preferential ketonase inhibitor (Table 1), on LPS-induced hypothermia in freely moving mice. This experimental model resembles the manifestation of severe forms of systemic inflammation (e.g. septic shock)^74^. We found that pre-treatment of the mice with compound (**24**) augmented the hypothermic response to high-dose LPS in mice, suggesting that the enzymatic, mostly ketonase, activity of MIF plays a limiting role in the thermoregulatory changes associated with severe forms of systemic inflammation.

Circulating inflammatory cytokines are known to be involved in the mediation of endotoxin-induced fever and hypothermia^74^^,^^81^^,^^83^, but the key cryogenic substances responsible for triggering the hypothermic response in severe systemic inflammation are still not entirely identified. LPS binds to toll-like receptor 4, which results in the activation of the NF-κB, thereby leading to the expression of proinflammatory cytokines such as tumour necrosis factor (TNF)-α, interleukin (IL)-1ß, and IL-6. At moderate-to-high concentrations TNF-α causes hypothermia, and, at present, it is considered to be the most important cryogenic mediator, whereas IL-6 is the most important pyrogenic cytokine^74^. MIF, as a potent proinflammatory cytokine, also plays an important role in systemic inflammation^84^, however, to our knowledge; it has not been clarified whether it also contributes to the thermoregulatory manifestations of the disease. In the present study, we demonstrated, for the first time, that LPS hypothermia is more pronounced when the ketonase (and possibly, to lesser extent, the enolase) activity of MIF is inhibited. These findings suggest that the enzymatic activity of MIF limits the effects of the endogenous, thermally active mediators, e.g. IL-6 and TNF-α, in severe systemic inflammation. In accordance, MIF tautomerase inhibitors blocked the production of TNF-α and to a bigger extent that of IL-6 in infected mice *in vivo*^85^, as well as, in LPS-activated macrophages *in vitro* in the present study (Figure 7). Compound (**24**) inhibits the ketonase activity of MIF with a five-fold higher potency than its enolase activity, but it cannot be ruled out that the enolase activity was also inhibited by the applied dose of compound (**24**) to some extent, therefore, that the enolase activity of MIF also plays a role in the control of the systemic inflammation-associated hypothermic response. The contribution of MIF and its enzymatic activities to the thermoregulatory manifestation of mild and moderate forms of systemic inflammation, which are typically accompanied by fever, also remain subject of future studies.

In conclusion, we have selected two families of compounds, *E*-2-arylmethylene-1-tetralones and their heteroanalogues as potential MIF inhibitors. The type and the size of the B-ring and the substitution pattern of the C-ring have a high impact on the bioactivity. From the compounds with unsubstituted C-ring the best inhibitor of the ketonase was the five membered indanone derivative (**1**). As regards the tetralone derivatives with homoaromatic rings the *p*-methyl derivative (**4**) showed the highest activity. As for the heteroaromatic C-rings, the five-membered derivatives as the indolyl- (**23**), the furyl derivative (**9**) and *N*-metylpyrrolyl compound (**22**) proved to be the most efficient. From the derivatives with six membered heteroaromatic C-ring, the 2-pyridyl substance (**24**) was the most efficient. The inhibitory effect of the enolase was much weaker. Some test substances, such as (**4**), *p*-methyl derivative possessed high selectivity. The possible mechanism of action is probably based on a Michael addition between the enone groups of the test compounds and the Pro1 of MIF. The selected five compounds (**4**), (**23**), (**24**), (**26**) and (**32**) inhibited LPS-induced macrophage activation, but to different extent. The pyridyl-derivatives (**24**) and (**26**) showed the highest potency, which might underline the importance of six membered heteroaromatic C-ring in the biological efficacy of our MIF inhibitors. We have determined a binding model for the compounds through docking experiments, and generated a custom covalent docking protocol, which we used to provide the structure of the hypothesised covalent adduct with Pro1. Through MD simulations, we have demonstrated that the determined reversible binding mode is stable, and covalent reaction between Pro1 and the electrophilic methylene carbon may be possible. Our work provide useful information for further understanding the ligand binding of MIF, and could be of aid in designing more potent inhibitors.

## Supplementary Material

Supplemental Material
