# Supplementary figures and images for: Tetralone derivatives are MIF tautomerase inhibitors and attenuate macrophage activation and amplify the hypothermic response in endotoxemic mice
Source: J Enzyme Inhib Med Chem. 2021 Jul 6;36(1):1357–69. doi: 10.1080/14756366.2021.1916010 (PMC8266241; doi:10.1080/14756366.2021.1916010)

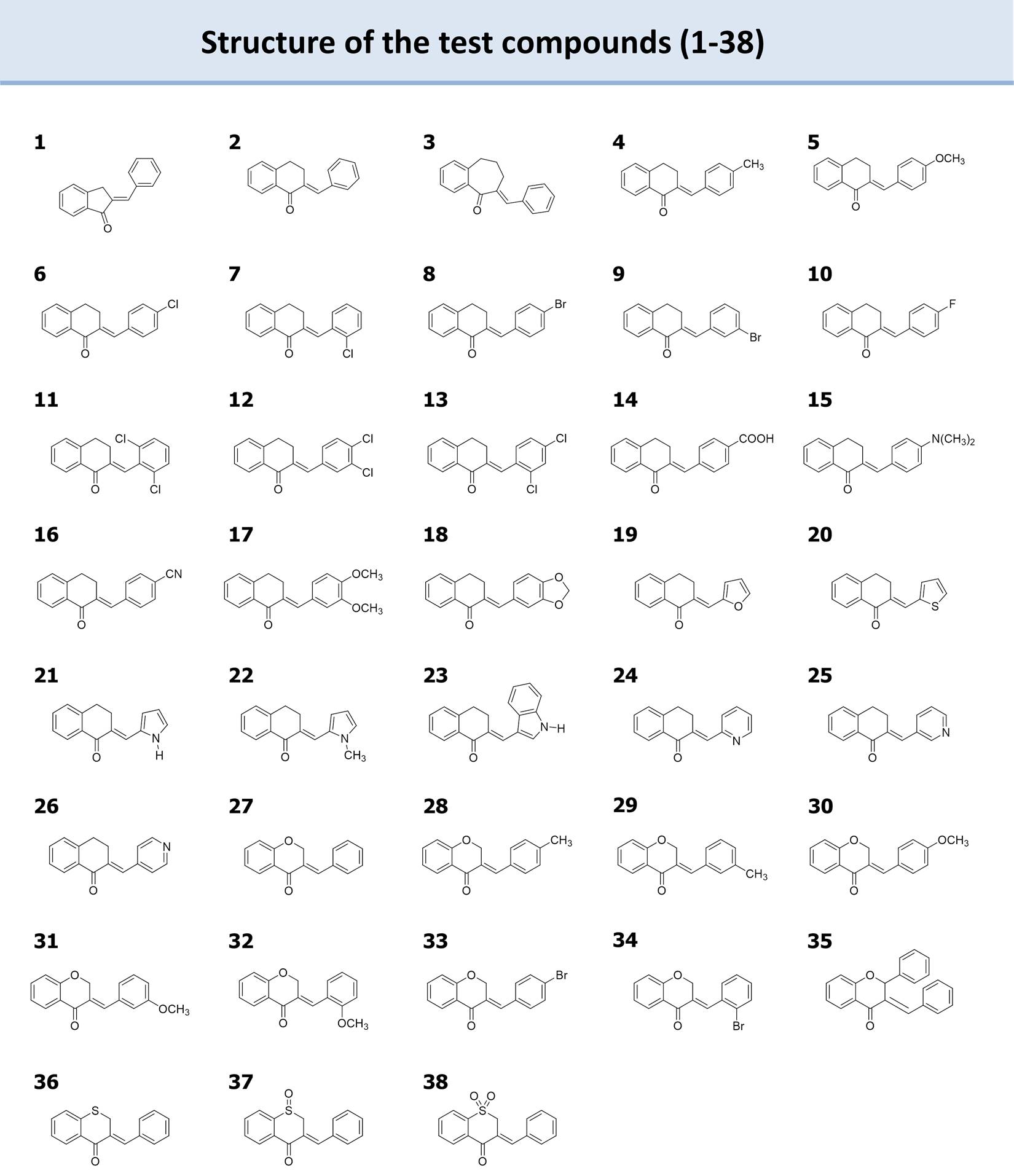

Supplement: Supplemental Material [file IENZ_A_1916010_SM9564.zip › Supp Material Fig S1 Structure of the test compounds.jpg]

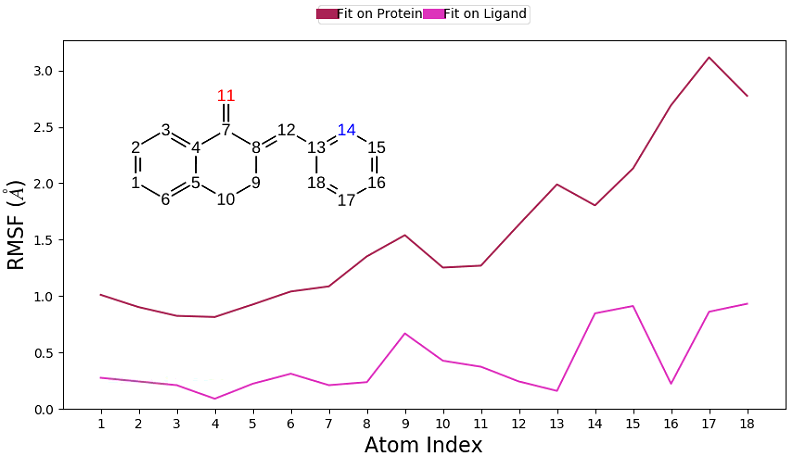

Supplement: Supplemental Material [file IENZ_A_1916010_SM9564.zip › Supp Material Fig S2 L RMSF 175ns.tif]

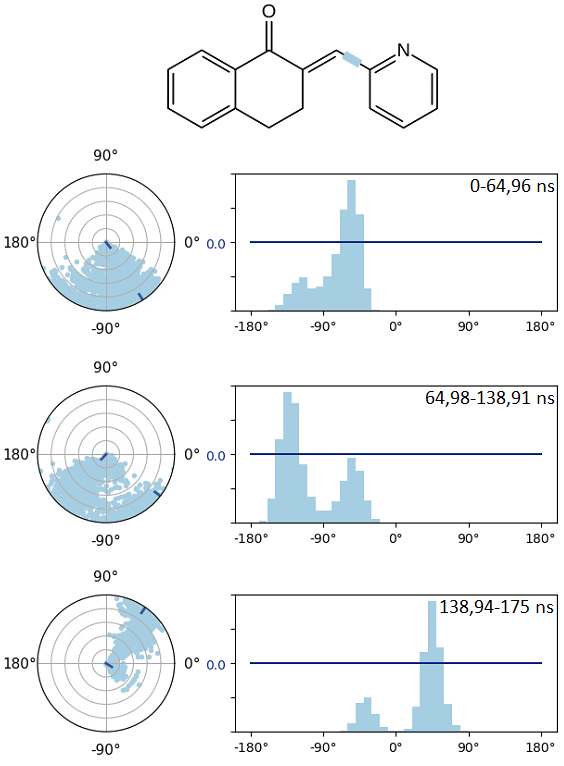

Supplement: Supplemental Material [file IENZ_A_1916010_SM9564.zip › Supp Material Fig S3 L Torsions.tif]

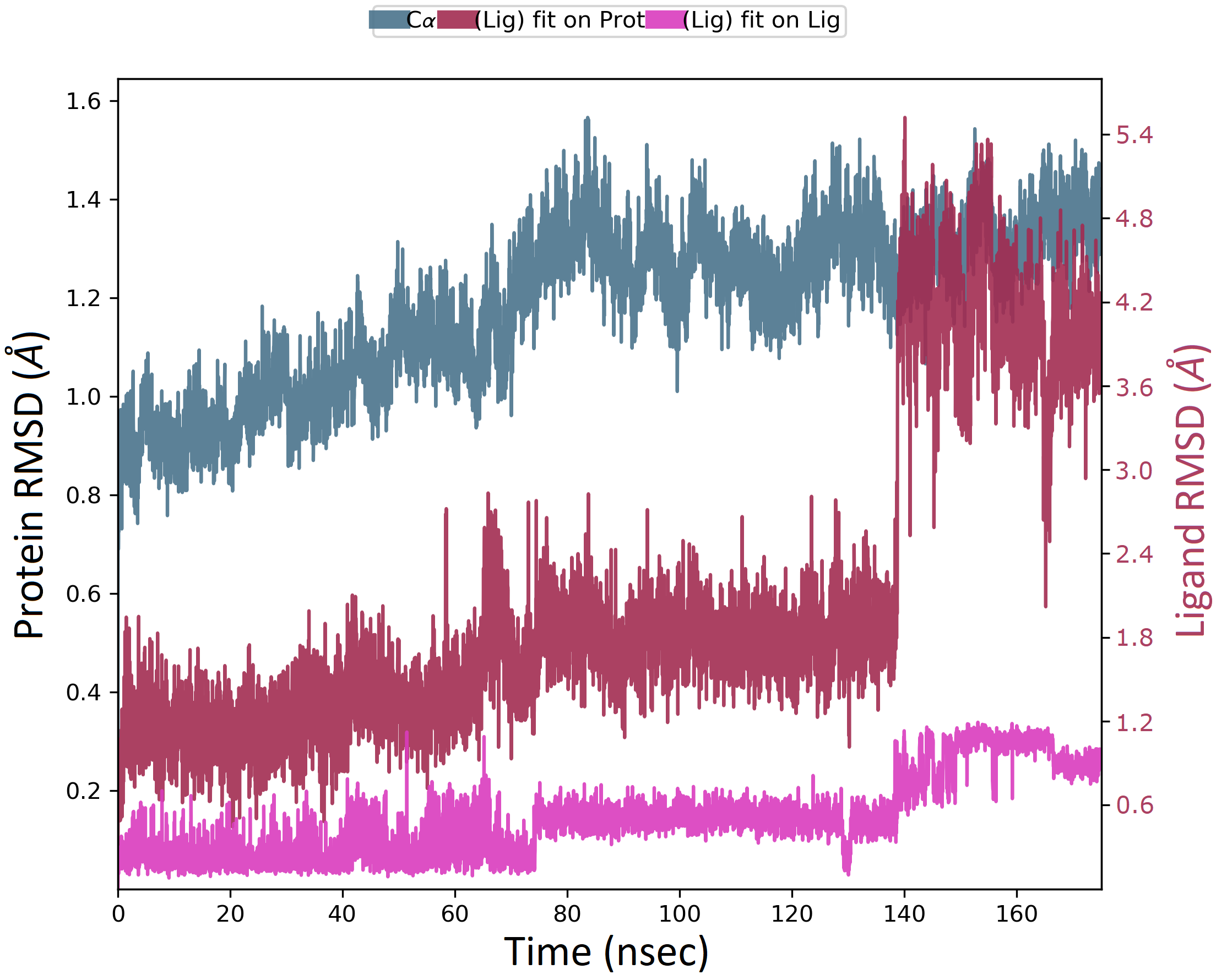

Supplement: Supplemental Material [file IENZ_A_1916010_SM9564.zip › Supp Material Fig S4 PL RMSD 175ns.tif]

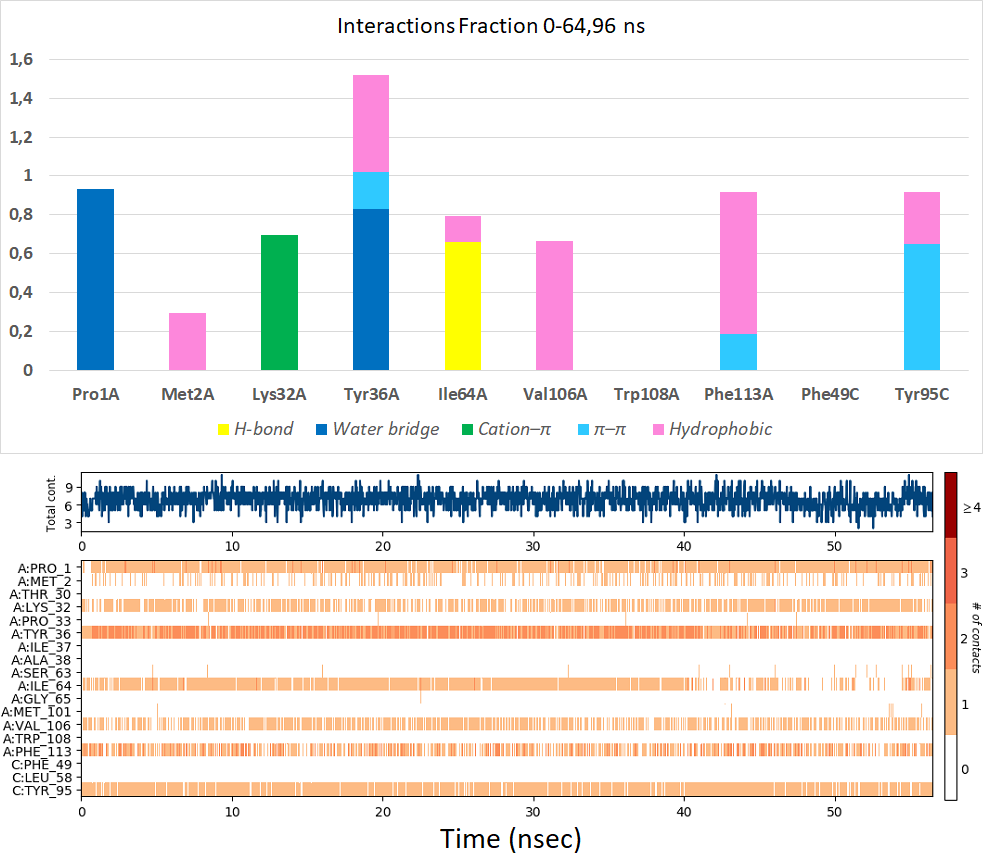

Supplement: Supplemental Material [file IENZ_A_1916010_SM9564.zip › Supp Material Fig S5 PL Contacts_1.tif]

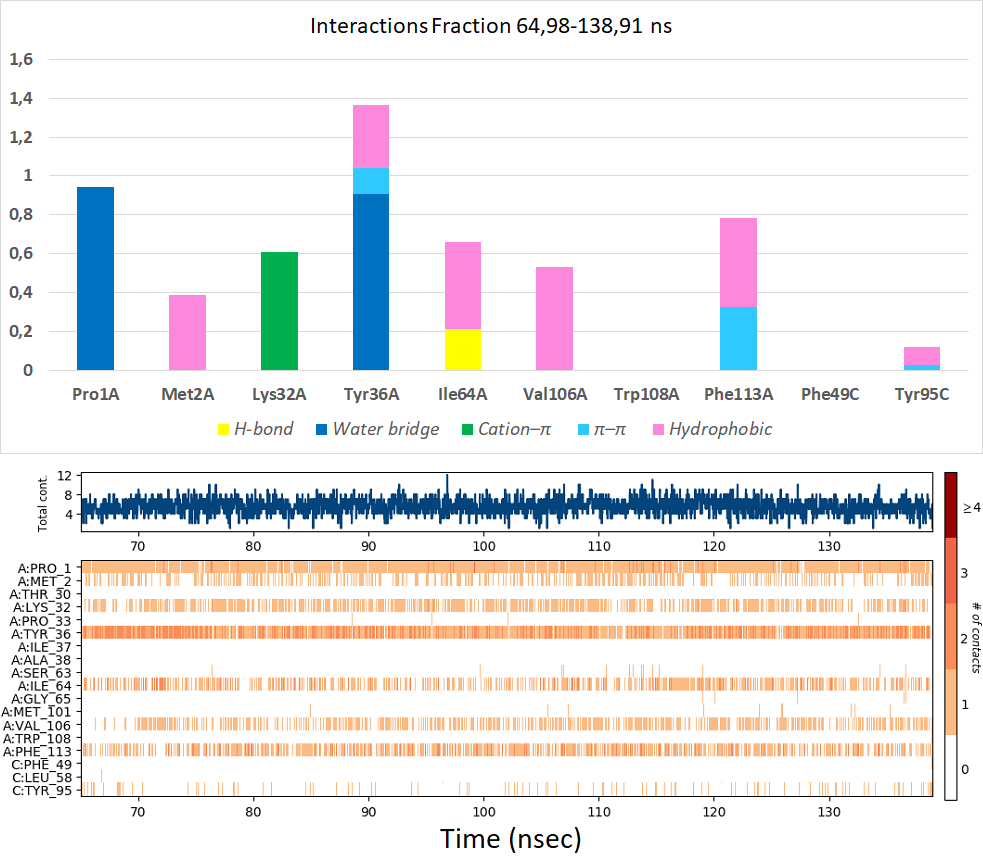

Supplement: Supplemental Material [file IENZ_A_1916010_SM9564.zip › Supp Material Fig S6 PL Contacts_2.tif]

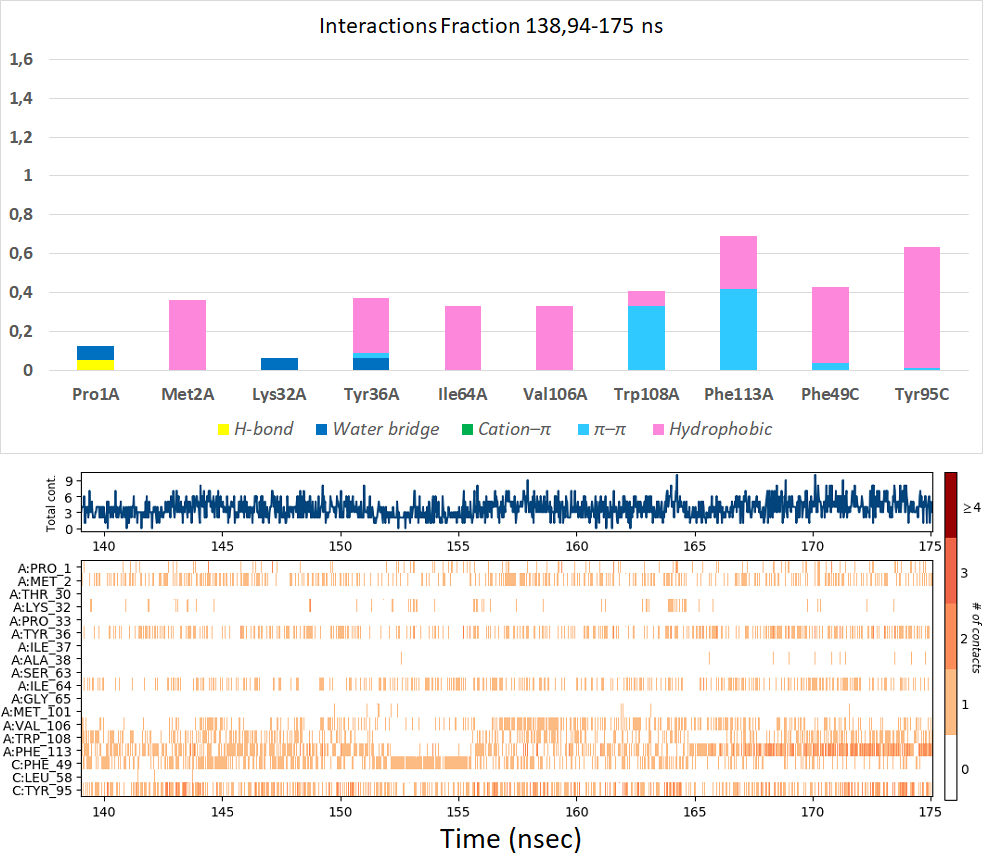

Supplement: Supplemental Material [file IENZ_A_1916010_SM9564.zip › Supp Material Fig S7 PL Contacts_3.tif]
